# Editorial: The impact of the human genome on interindividual variability in drug response

**DOI:** 10.3389/fgene.2024.1401604

**Published:** 2024-04-18

**Authors:** Yaya Kassogue, Ingrid Fricke-Galindo

**Affiliations:** ^1^ Centre de Recherche et de Formation sur les Pathologies Moléculaires, Faculté de Médecine et d’Odonto-Stomatologie, Université des Sciences, des Techniques et des Technologies de Bamako, Mali, West Africa; ^2^ HLA Laboratory, Instituto Nacional de Enfermedades Respiratorias Ismael Cosio Villegas, Mexico City, Mexico

**Keywords:** pharmacogenetics, pharmacogenomics, drug response, interindividual variability, genetics

The genetic background of patients contributes to the variability in drug metabolism and response. Several genetic variants can be found in genes coding drug-metabolizing enzymes, transporters, receptors, and other drug-interacting proteins (Meyer, 2003; Hilli et al., 2007). Over the years, the pharmacogenetic information has exponentially increased, and other inquiries have been raised. For instance, the differences in the frequencies of the biomarkers according to the ancestry of the studied population and the necessity of the meta-analysis have been addressed (Bertilsson, 1995).

This Research Topic aims to improve our understanding of how individual genetic variants affect therapy response. We were particularly interested in understanding the impact of genetic inheritance on the treatment response of a variety of disorders, including infectious, malignant, cardiovascular, endocrine, pulmonary, digestive, rheumatological, neurological, ocular, hematological, mental, and autoimmune.

The Research Topic was open to any work closely examining the impact of genetic background on interindividual variability in treatment by exploring different diseases, using candidate gene or whole genome approaches. The rigor applied to peer reviews enabled us to consider four for publication in this Research Topic.

In agreement with the recognized inter-ethnic variation in the frequency of pharmacogenomic biomarkers, the first paper accepted comprised a Comprehensive characterization of pharmacogenes in a Taiwanese Han population Lu et al. This study aimed to fill a gap in the distribution of clinically relevant pharmacogenes in the East Asian population. A total of 14 useable pharmacogenomic diplotypes and phenotypes in around 172,854 individuals were analyzed based on medical record data. The authors found that the frequencies of PGx phenotypes were comparable to those in East Asia. They also noted that 99.9% of participants had at least one actionable pharmacogenetic phenotype. Interestingly, their analysis revealed that 29% of participants had taken a drug likely to cause an inadequate response. This study clearly shows the importance of prior knowledge of pharmacogenetic data before starting treatment.

The remaining manuscripts deal with relevant pharmacogenomic biomarkers in the CYP450 complex. The second paper was titled A frequent CYP2D6 variant promotes skipping of exon 3 and reduces CYP2D6 protein expression in human liver samples Collins et al. In this study, the authors used fragment analysis and RT-qPCR to understand the impact of rs1058164 G (MAF = 27%–43%) on CYP2D6∆E3 expression in human liver samples and transfected cells; and noted a 1.4 to 2.5-fold increase in CYP2D6∆E3 formation. In addition, they performed a western blots assay which showed that the SNP could account for a 50% decrease in full-length hepatic CYP2D6 protein expression. The study investigators found that rs16947 (CYP2D6*2) decreases full-length CYP2D6 mRNA by boosting the synthesis of an unstable splice isoform lacking exon 6 (CYP2D6ΔE6). However, the impact of CYP2D6ΔE6 is countered in carriers of the rs5758550 downstream enhancement variant. The authors conclude that clinical studies are needed to better understand the influence of haplotypes formed by these three SNPs for an improved prediction alongside the current standard of CYP2D6 models.

As a source of accurate and useful information, the third manuscript consists of A meta-analysis of the pooled impact of CYP7A1 single nucleotide polymorphisms on serum lipid responses to statins Lim et al. Here, the authors tested the relationship between the effect of statins and cholesterol levels in studies involving participants with the *CYP7A1* variant. The meta-analysis included six articles with a total of 1,686 subjects (cholesterol, LDL-C, and HDL-C) and 1,156 subjects (triglycerides), selected after carefully checking scientific databases such as PUBMED, Cochrane, and EMBASE. A greater reduction in total cholesterol was noted in the non-carriers of a *CYP7A1* SNP (−204 A/C (rs3808607), −278 A/C (rs3808607) and rs8192875). In sum, the authors conclude that the variant allele of *CYP7A1* may lead to suboptimal control of total cholesterol and LDL-C levels when a statin is administered.

Finally, the fourth paper was Influence of cytochrome P450 2D6*10/*10 genotype on the risk for tramadol associated adverse effects: a retrospective cohort study Mahajna et al. The authors of this retrospective investigation used a binary logistic regression model to explore the link between the CYP2D6*10/*10 genotype and long-term tramadol adverse effects. Of the 493 participants, 5.1% were heterozygous, while 11% carried the CYP2D6*10/*10 genotype. The authors discovered that participants with the CYP2D6*10/*10 variant had a greater risk of adverse outcomes, notably in the central nervous and gastrointestinal systems. Tramadol long-term use requires upstream pharmacogenetic information to guarantee that the drug is both tolerable and effective, hence increasing patients’ quality of life.

These accepted articles have highlighted the value of individual genetic information in medical treatment results, paving the path for pharmacogenetic evidence-based patient management.
